# Comparative short-term safety of bolus versus maintenance iron dosing in hemodialysis patients: a replication study

**DOI:** 10.1186/1471-2369-15-154

**Published:** 2014-09-22

**Authors:** Janet K Freburger, Alan R Ellis, Abhijit V Kshirsagar, Lily Wang, M Alan Brookhart

**Affiliations:** Cecil G. Sheps Center for Health Services Research, University of North Carolina, 725 Martin Luther King, Jr. Blvd., CB# 7590, Chapel Hill, NC 27599-7590 North Carolina; UNC Kidney Center, University of North Carolina, 7024 Burnett-Womack, CB# 7155, Chapel Hill, NC 27599-7155 North Carolina; Department of Epidemiology, University of North Carolina, 2101 McGavran-Greenberg Hall, CB# 7435, Chapel Hill, NC 27599-7435 North Carolina

**Keywords:** Hemodialysis, Intravenous iron, Anemia management

## Abstract

**Background:**

Recent research has reported that patients receiving bolus (frequent large doses to achieve iron repletion) versus maintenance dosing of iron have an increased short-term risk of infection, but a similar risk of cardiovascular events. We sought to determine whether these findings could be replicated using the same methods and a different data source.

**Methods:**

Clinical data from 6,605 patients of a small U.S. dialysis provider merged with Medicare claims data were examined. Iron dosing patterns (bolus, maintenance, no iron) were identified during 1-month exposure periods and cardiovascular and infection-related outcomes were assessed during 3-month follow-up periods. The effects of bolus versus maintenance dosing were assessed using Cox proportional hazards regression analyses to estimate hazard ratios and semiparametric additive risk models to estimate hazard rate differences, controlling for demographic and clinical characteristics, laboratory values and medications, and comorbidities.

**Results:**

48,050 exposure/follow-up periods were examined. 13.9 percent of the exposure periods were bolus dosing, 49.3 percent were maintenance dosing, and the remainder were no iron use. All of the adjusted hazard ratios were >1.00 for the infection-related outcomes, suggesting that bolus dosing increases the risk of these events. The effects were greatest for hospitalized for infection of any major organ system (hazard ratio 1.13 (1.03, 1.24)) and use of intravenous antibiotics (hazard ratio 1.08 (1.02, 1.15). When examining the subgroup of individuals with catheters, the hazard ratios for the infection-related outcomes were generally greater than in the overall sample. There was little association between type of dosing practice and cardiovascular outcomes.

**Conclusions:**

Results of this study provide further evidence of the association between bolus dosing and increased infection risk, particularly in the subgroup of patients with a catheter, and of the lack of an association between dosing practices and cardiovascular outcomes.

**Electronic supplementary material:**

The online version of this article (doi:10.1186/1471-2369-15-154) contains supplementary material, which is available to authorized users.

## Background

Intravenous iron is used in combination with erythropoiesis-stimulating agents (ESAs) to treat the anemia of hemodialysis patients. Recent studies have reported a beneficial effect of iron dosing on anemia parameters in hemodialysis patients [1–4]. While the safety of ESAs in chronic kidney disease has been examined in clinical trials [5–7], less is known about the safety of intravenous iron. Several biological mechanisms suggest that sub-optimal use of iron could lead to adverse clinical events [8]. Frequent administration of iron may lead to oversaturation of transferrin and the release of free, catalytically active iron into the plasma [9]. Because iron is essential for bacterial growth, free iron in circulation may increase the risk of infection [10, 11]. Indeed, frequency of iron administration has been found to be associated with increased risk of infection-related mortality in ESRD [12]. Free iron is also known to catalyze the formation of highly reactive oxygen species [13, 14]. These could give rise to lipid radicals, which may damage tissue [15] and lead to atherogenesis [16], possibly increasing the risk of cardiovascular events [8, 17].

A recently completed a large-scale observational study examined the short-term comparative safety of intravenous iron dosing strategies in hemodialysis patients and found that patients receiving bolus (frequent large doses to achieve iron repletion) versus maintenance dosing had higher risks of infection-related hospitalization and infection-related death, and that these risks were greatest among the subgroup of patients with a dialysis catheter [18]. No association was found between large-dose intravenous iron treatment strategies and cardiovascular morbidity and mortality [19]. The study was conducted using data from a large U.S. dialysis chain merged with data from the United States Renal Data System (USRDS). Because the previous study was the first large epidemiologic study to address risks of adverse events associated with intravenous iron use, we sought to determine whether these findings could be replicated using the same methods with data from another U.S. dialysis chain where the patient demographics, case mix, and other aspects of medical practice may differ. Specifically, we examined short-term infection and cardiovascular risk associated with bolus versus maintenance intravenous iron dosing in a cohort of patients undergoing chronic hemodialysis.

## Methods

### Data sources

Data for this study came from the clinical database of a small U.S. dialysis organization and the United States Renal Data System (USRDS), a national data system that collects, analyzes and distributes information about the treatment of ESRD. The clinical database contains information on approximately 6,000 dialysis patients per year from approximately 60 dialysis facilities, primarily located in the Northeast and Midwest. Clinical, laboratory, and treatment data are captured using standardized electronic data entry. We used the clinical database to obtain detailed information on iron dosing, epoetin alfa (EPO) dosing, clinical laboratory values (e.g., hemoglobin, transferrin saturation [TSAT], serum ferritin), and current vascular access. These data were merged with data from the following USRDS files: the Medical Evidence Report Form, the Medicare Enrollment files, and the standard analytic files, which contain final action Medicare claims [20]. Data extracted from the USRDS files included demographic, health care use (e.g., hospitalizations, outpatient care), comorbidity, and clinical (e.g., vintage) information. Both the clinical data and USRDS data were purchased by the study team and were governed by Data Use Agreements with the Renal Research Institute and the USRDS, respectively. These data are not freely available to other researchers.

### Study design

We utilized a retrospective cohort design with a 6-month baseline period, a one-month iron and EPO exposure period, and a three-month follow-up period. The index date of the exposure period was anchored on a TSAT lab as this information is used to guide iron administration. While serum ferritin labs may also guide iron administration, we focused on TSAT labs only because these were, on average, measured monthly in our data, whereas serum ferritin labs occurred less frequently (every 2–3 months on average). The exposure period began the day after the qualifying TSAT measurement.

**Figure 1:**
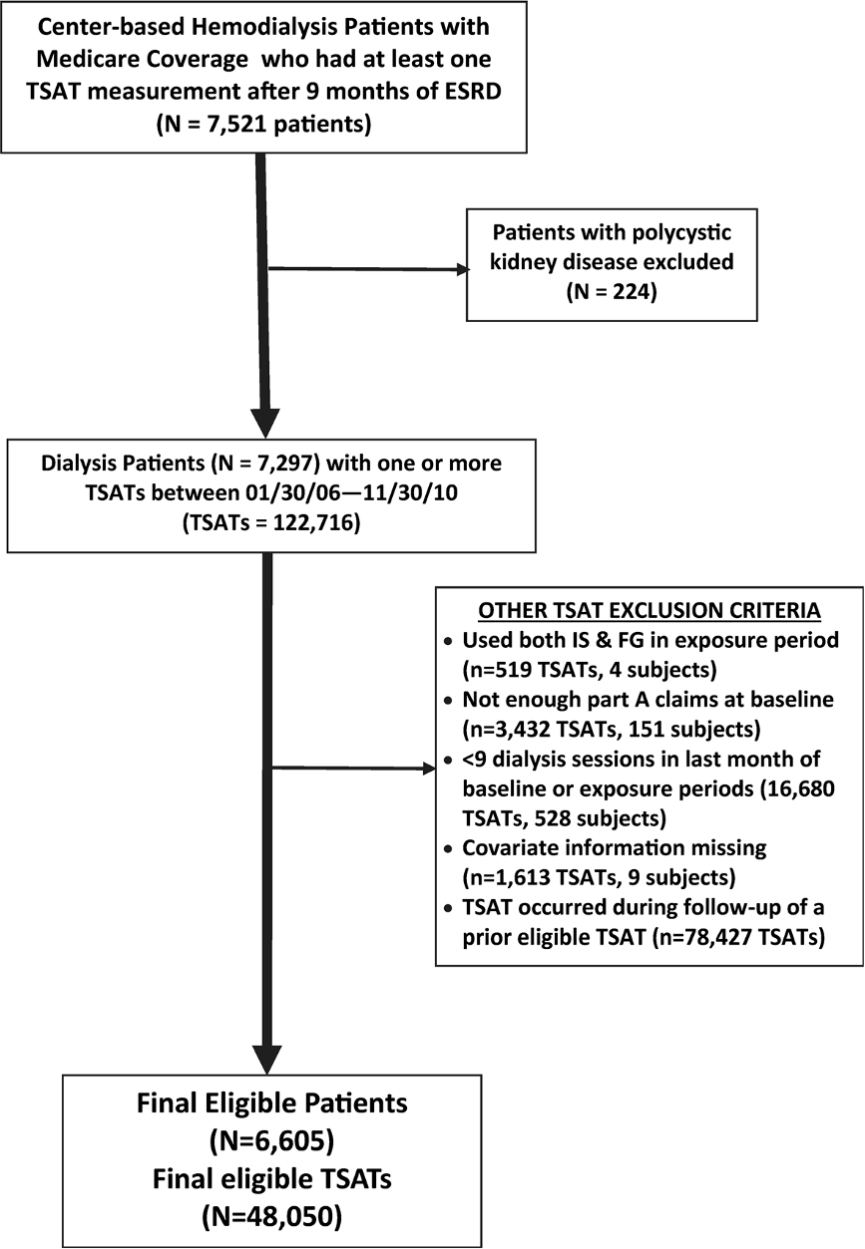
**Cohort creation.**

### Cohort identification

We first identified center-based hemodialysis patients who had at least one TSAT measurement after undergoing dialysis for at least 9 months. The 9-month period accounted for the 6-month baseline period and an additional 3 months from dialysis initiation to allow for stability in the CMS claims processing [20]. Individuals with polycystic kidney disease were then excluded as the anemia management of these patients differs greatly from that of most ESRD patients receiving hemodialysis. Eligible patients could contribute more than one TSAT measurement. TSAT measurements were eligible if they occurred between January 30, 2006 (to allow assessment of lab values and medications in the last month of baseline) and November 30, 2010 (to allow for the 1-month exposure period and at least one day of follow-up).

Measurements of TSAT were excluded if 1) both ferric gluconate and iron sucrose were administered during the exposure period; 2) there was an insufficient duration of Part A claims at baseline (i.e., <120 days of Part A claims), suggesting incomplete data; or 3) there were fewer than 9 dialysis sessions in the last month of baseline or during the exposure period. We also excluded TSAT records with missing covariate information and TSAT measurements that occurred in the follow-up period of a prior eligible TSAT (Figure 1).

### Study variables

#### Exposures

The primary iron exposures of interest were bolus versus maintenance iron dosing. We also created a no iron category for individuals who received no iron during the one month exposure period. A month was classified as a bolus month if, during that month, two consecutive iron doses of at least 100 mg were administered and the total iron dose had the potential to exceed 600 mg within 30 days based on spacing between the doses in the sequence. For example, two consecutive iron doses of 200 mg each, within 10 days, would qualify as a bolus dose according to our definition. Months that had no bolus dosing patterns were classified as “maintenance months”.

#### Outcomes

We examined eight adverse clinical outcomes: death from any cause; hospitalized for pneumonia, vascular access infection, or sepsis; hospitalized for infection of any major organ system; use of intravenous antibiotics; hospitalized for myocardial infarction (MI), hospitalized for stroke, and cardiovascular disease-related death. We also created three composite outcomes: hospitalized for pneumonia, vascular access infection, or sepsis or infection-related death (a more specific infection-related composite outcome); hospitalized for any infection or use of intravenous antibiotics (a more sensitive infection-related composite outcome); and cardiovascular hospitalization or cardiovascular-related death. Outcomes were determined by examining the Medicare death notification and Medicare inpatient and outpatient claims. The specific codes used to define our outcomes are presented in an additional online file (Additional file 1: Table S1).

#### Covariates

We included several covariates in our analyses (Additional file 1: Table S2) to control for potential confounding. The choice of these variables was based on content expertise of the study team, previous literature, and data availability and included demographic characteristics, clinical characteristics (e.g., vintage, BMI, type of vascular access), laboratory and anemia management variables (e.g., baseline hemoglobin, ferritin, TSAT, concurrent EPO dose), and hospital days. We also included several comorbidity measures (e.g., prior infections, prior cardiovascular events), based on the Elixhauser classification [21] and content expertise of the investigative team.

### Statistical analyses

To assess the relation between iron dosing practices and adverse outcomes, we used Cox proportional hazards regression analyses to estimate hazard ratios and semiparametric additive risk models [22] to estimate hazard rate differences. Additive models estimate the absolute difference in event rate per unit change in the exposure variable, whereas Cox models provide estimates of relative hazard on a multiplicative scale [22]. From a clinical and public health perspective, the absolute difference may be the more useful estimate because it provides information about the number of harmful or beneficial events for a given unit of change in the exposure over a given period of time. To account for the within-patient correlation of the repeated measures, we used the robust sandwich covariance estimate [23] for the Cox models and a cluster bootstrap estimation for the additive risk models. We addressed potential confounding by controlling for the covariates described above. Individuals were censored by death (for the hospitalization outcome), loss to follow-up, kidney transplant, or administratively by the end of available data. We conducted analyses on the entire sample and, based on results of the previous study where effects were greatest in the catheter subgroup [18], examined the infection-related outcomes in the subset of individuals who used a catheter. We did not conduct any further subgroup analyses because there was no evidence of other subgroup effects/effect modification in the previous study and due to sample size limitations.

#### Sensitivity analyses

We assessed the sensitivity of our results to the addition of other potentially relevant covariates (Additional file 1: Table S2) and to changes in the length of the exposure/follow-up periods: 1 month/6 weeks, 2 weeks/6 weeks, and 1 week/6 weeks (Additional file 1: Table S3). We also conducted a sensitivity analysis using a propensity score approach with inverse probability of treatment weights [24]. We used logit models to predict the iron categories (bolus, maintenance) among those who received iron. For each observation, we then estimated the probability of receiving the treatment actually received and took the reciprocal of this value to create the inverse-probability-of-treatment weights. The weights were then stabilized [24] and used in the Cox models for the full sample and the catheter subgroup using the 1 month exposure/3 month follow-up study design.

This study was reviewed and approved by the Institutional Review Board (Public Health-Nursing) at the University of North Carolina, Chapel Hill, NC (Study #10-1674) and was exempt from requiring patient consent. While the IRB reviewed the DUAs for the data used in this study, it did not have any direct influence on data accession.

## Results

6,605 patients met study entry requirements and contributed data on 48,050 exposure/follow-up periods (Figure 1). Sample characteristics are presented in Table 1, stratified by iron exposure group. Relative to the earlier study [18], the sample for this study had a greater proportion of Blacks and residents of the Northeast. Patients in this study were also on dialysis longer, had less catheter use, a higher proportion of comorbidities, and higher rates of infection at baseline. Demographic and clinical characteristics were generally similar among the dosing groups. Catheter use was highest in the bolus dosing group and lowest in the no iron group. TSAT and ferritin values were lowest in the bolus group and highest in the no iron group, as might be expected. The prevalence of comorbidities was generally highest in the bolus dosing group and lowest in the no iron group.

**Table 1:** **Demographics and clinical characteristics of sample, stratified by iron dosing (N=48,050)**

Characteristics, mean (SD) or%	Bolus (13.9%)	Maintenance (49.3%)	Non-user (36.8%)
*Demographic*			
Age, y	60.1 (15.3)	61.4 (15.1)	61.4 (15.1)
Female	45.8	45.1	45.3
Race: White	38.2	40.1	37.3
Black	56.7	54.1	56.8
Other	5.1	5.7	5.9
Region: Midwest	6.2	11.0	8.1
Northeast	80.6	84.3	78.6
South	10.6	2.8	9.6
West	1.8	1.4	3.0
*Clinical*			
Vintage, y	4.8 (4.7)	5.1 (5.0)	5.8 (5.5)
Body Mass Index	32.8 (23.5)	33.9 (29.8)	31.6 (24.7)
Catheter Use	21.6	19.5	17.2
*Laboratory and Anemia Management Variables*			
Albumin at baseline	3.84 (0.41)	3.91 (0.37)	3.93 (0.38)
Hemoglobin at baseline	11.5 (1.4)	12.0 (1.3)	11.9 (1.4)
Index TSAT	23.7 (9.5)	31.4 (10.9)	35.8 (14.3)
Ferritin at baseline	625 (479)	745 (536)	862 (588)
Iron (mg) at baseline	314 (316)	279 (214)	109 (241)
Iron (mg) during exposure	700 (291)	227 (118)	0.0 (0.0)
EPO at baseline (1000U)	111 (98)	75.3 (78.3)	62.0 (74.0)
EPO during exposure (1000U)	113 (100)	73.1 (77.0)	63.0 (73.9)
*Comorbidities*			
Hospital days in last month	1.0 (2.3)	0.6 (1.9)	0.5 (1.8)
Infection in last month	17.5	12.6	10.4
Infection in last 6 mos. Pneumonia	16.7	12.8	11.5
Sepsis	23.5	19.0	14.9
Vascular access	15.0	10.1	9.2
Diabetes	67.5	64.1	60.7
Ischemic stroke	16.7	12.9	12.5
Myocardial Infarction	6.2	4.4	4.0
COPD, Asthma	24.4	20.0	18.0
Cancer	12.1	10.9	11.7
GI bleeding	9.5	6.1	5.2

**Figure 2:**
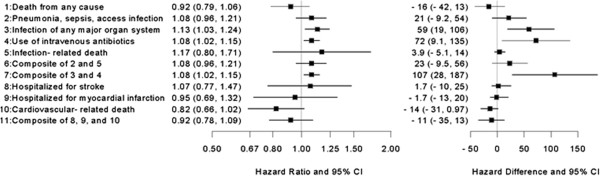
**Forest plot of study outcomes for the entire sample (N=48,050).** Forest plot of multivariable adjusted hazard ratios and rate differences between bolus versus maintenance (referent) dosing and study outcomes for the entire sample.

Table 2 presents the adjusted hazard ratios and rate differences for the comparison of bolus versus maintenance dosing for the entire sample. These findings are graphically represented in Figure 2. All of the adjusted hazard ratios were >1.00 for the infection-related outcomes, though the 95% confidence intervals included 1.00 for three of the six hazard ratios. The effects were greatest for hospitalized for infection of any major organ system (HR: 1.13) and use of intravenous antibiotics (HR: 1.08) and indicate that bolus dosing increases the risk of these events. On the additive scale, individuals who received bolus dosing had 59 more hospitalizations for major organ system infection, per 1000 person-years, than individuals who received maintenance dosing. Use of intravenous antibiotics was also higher among individuals who received bolus versus maintenance dosing (72 more events per 1000 person-years).

**Table 2:** **Multivariable adjusted associations between bolus versus maintenance (Referent) dosing and study outcome (N=48,050)**

Outcome	Effect measure ^1^	Estimate (95% CI)
Death from any cause	Hazard Ratio	0.92 (0.79, 1.06)
	Rate Difference	-16 (-42, 13)
***Infection Outcomes***		
Hospitalized for pneumonia, sepsis, vascular access infection	Hazard Ratio	1.08 (0.96, 1.21)
	Rate Difference	21 (-9.2, 54)
Hospitalized for infection of any major organ system	Hazard Ratio	1.13 (1.03, 1.24)
	Rate Difference	59 (19, 106)
Use of intravenous antibiotics	Hazard Ratio	1.08 (1.02, 1.15)
	Rate Difference	72 (9.1, 135)
Infection-Related Death	Hazard Ratio	1.17 (0.80, 1.71)
	Rate Difference	3.9 (-5.1, 14)
Hospitalized for pneumonia, vascular access infection, sepsis or infection-related death	Hazard Ratio	1.08 (0.96, 1.21)
	Rate Difference	23 (-9.5, 56)
Hospitalized for any infection or use of intravenous antibiotics	Hazard Ratio	1.08 (1.02, 1.15)
	Rate Difference	107 (28, 187)
***Cardiovascular Outcomes***		
Hospitalized for stroke	Hazard Ratio	1.07 (0.77, 1.47)
	Rate Difference	1.7 (-10, 15)
Hospitalized for Myocardial Infarction	Hazard Ratio	0.95 (0.69, 1.32)
	Rate Difference	-1.7 (-13, 10)
Cardiovascular-related death	Hazard Ratio	0.82 (0.66, 1.02)
	Rate Difference	-14 (-31, 0.97)
Cardiovascular-related hospitalization or death	Hazard Ratio	0.92 (0.78, 1.09)
	Rate Difference	-11 (-35, 13)

^1^Rate difference per 1000 patient years.

The adjusted hazard ratios for all-cause death and cardiovascular outcomes were relatively imprecise, with confidence intervals including the null. Relative to the rate differences for the infection outcomes, the rate differences for all-cause death and the cardiovascular outcomes were much closer to the null, though there was a slight suggestion of a protective effect on cardiovascular death (Table 2 and Figure 2). Our results were robust to variations in the length of the exposure and follow-up periods (Additional file 1: Table S3) and the addition of covariates beyond our *a priori* specified multivariable models (Additional file 1: Table S4).

**Figure 3:**
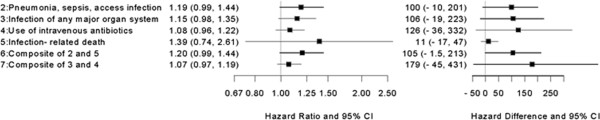
**Forest plot of infection-related outcomes for the catheter subgroup (N=9,113).** Forest plot of multivariable adjusted associations between bolus versus maintenance (referent) dosing and infection-related outcomes for the catheter subgroup.

The adjusted hazard ratios and rate differences for the infection-related outcomes in the catheter subgroup are presented in Table 3 with graphic representation in Figure 3. The effect measures all indicated increased risk, but were imprecise, with wide 95 percent confidence intervals that included the null. Three of the hazard ratios approached statistical significance, with the lower limit of the confidence interval 0.98 or 0.99.

**Table 3:** **Multivariable adjusted associations between bolus versus maintenance (Referent) dosing and infection-related outcomes for the catheter subgroup (N=9,113)**

Outcome	Effect measure ^1^	Estimate (95% CI)
**Hospitalized for pneumonia, sepsis, vascular access infection**	Hazard Ratio	1.19 (0.99, 1.44)
	Rate Difference	100 (-10, 201)
**Hospitalized for infection of any major organ system**	Hazard Ratio	1.15 (0.98, 1.35)
	Rate Difference	106 (-19, 223)
**Use of intravenous antibiotics**	Hazard Ratio	1.08 (0.96, 1.22)
	Rate Difference	126 (-36, 332)
**Infection-Related Death**	Hazard Ratio	1.39 (0.74, 2.61)
	Rate Difference	11 (-17, 47)
**Hospitalized for pneumonia, vascular access infection, sepsis or infection-related death**	Hazard Ratio	1.20 (0.99, 1.44)
	Rate Difference	105 (-1.5, 213)
**Hospitalized for any infection or use of intravenous antibiotics**	Hazard Ratio	1.07 (0.97, 1.19)
	Rate Difference	179 (-45, 431)

^1^Rate difference per 1000 patient years.

Results of our analyses using IPTWs are presented in Additional file 1: Table S5 and Additional file 1: Table S6. The IPTWs balanced the sample characteristics for the bolus and maintenance groups with absolute standardized differences ranging from 0 – 0.15 with all differences < =0.05 with the exception of baseline iron which was 0.15. (Additional file 1: Table S5). These findings indicate that the weighting was successful in balancing the bolus and maintenance groups on baseline characteristics. The mean of the stabilized weights was 1.00, as expected [25], and there were no extremely small or large weights (0.25 – 9.1). A comparison of the results of the multivariable and IPTW-adjusted analyses is presented in Additional file 1: Table S6. For the full sample, there was little difference in the multivariate adjusted and IPTW adjusted results, particularly in regard to the infection outcomes. For the catheter subgroup, the point estimates for the two methods differed slightly more, relative to the full sample, and were much less precise. In most cases the point estimates were in the same direction for both analyses.

## Discussion

Consistent with previous work [18, 19], we found evidence of an association between bolus dosing and infection outcomes and no association between bolus dosing and cardiovascular outcomes, in a sample of patients receiving center-based hemodialysis. Patients receiving bolus versus maintenance dosing were more likely to have infection-related events, with the most notable rate differences for use of intravenous antibiotics and hospitalization for any infection. While some of the hazard ratios for the infection outcomes had confidence intervals that included the null, all of these hazard ratios were >1.00 (HR’s ranging from 1.08-1.17) and similar in magnitude to the hazard ratios in the previous study examining infection risks [18] (HR’s ranging from 1.05-1.11). All of the hazard ratios for the cardiovascular outcomes included the null, with one greater than 1.00 and the others less than 1.00. These hazard ratios ranged from 0.82-1.07, similar to those in the previous study examining cardiovascular risks [19] (HR’s ranging from 0.98-1.05).

The effect measures for the catheter subgroup (Table 3) were imprecise due to small sample size, but the magnitude of the rate differences suggests that this group, in particular, may be at greater risk of infection-related outcomes with bolus versus maintenance dosing. Of particular note are the rate differences for hospitalization for pneumonia, sepsis, or vascular access infection and the combination of these outcomes with infection-related death. Patients with a catheter who received bolus dosing experienced approximately 100 more events per 1000 person-years for these outcomes relative to patients with a catheter who received maintenance dosing. These findings also agree with the earlier study, which reported rate differences of approximately 75 more infection-related events per 1000 person-years when comparing bolus versus maintenance dosing in this subgroup [18].

Using the identical epidemiologic design and statistical methods as in the previous research, we examined the comparative short-term safety of bolus versus maintenance dosing using data on a different sample of U.S. center-based hemodialysis patients. While the sample for this analysis was much smaller (N = 48,050) than in the previous study (N = 776,203), our findings were remarkably similar. The characteristics of the two cohorts were similar in some respects, but differed in regard to some demographic and clinical characteristics. Also, a majority of patients in the current study were from the Northeastern part of the U.S., while most patients in the previous study resided in the South. The proportion of individuals that received bolus versus maintenance dosing in the two studies were remarkably similar (13.9% received bolus dosing in this study vs 12.6% and 49.3% received maintenance dosing vs 49.2%). While not particularly innovative, replicating studies provide important scientific information that may add to a body of evidence or call into question previous findings. Our study has several limitations, including its non-experimental design, its focus on short-term events only, and the potential for unmeasured confounding. We did examine our results with the addition of more covariates and found little change in the effect measures (Additional file 1: Table S3), suggesting that we adequately controlled for confounding due to measured covariates. Another limitation is that our study design required survival for at least 9 months following the start of dialysis, which limits the generalizability of our findings to incident hemodialysis patients. We also did not validate the categorization of our exposure variable against a gold standard. Rather, we examined the patterns and amount of iron dosing and used clinical expertise to arrive at our method of categorization.

Strengths of our study include the sound design, in which baseline covariates were identified before the exposure period and outcomes were ascertained after the exposure period, and our rich data base, which contained clinical data merged with administrative health care claims data. Building on earlier analyses, the results of this study provide further evidence of the potential risks of bolus dosing of intravenous iron. These risks should be considered in light of the reported benefits of bolus dosing, which include diminished ESA requirements and improved anemia management [1–4].

## Conclusions

We examined the short-term comparative safety of bolus versus maintenance dosing in a sample of center-based hemodialysis patients and found that bolus dosing was associated with increased infection risk, particularly in the subgroup of patients with a catheter. We found no association between dosing practices and cardiovascular outcomes.

## Electronic supplementary material

Additional file 1: Table S1: Adverse Study Outcomes. **Table S2.** Definition of Covariates. **Table S3.** Unadjusted and Multivariable Adjusted Associations1 Between Bolus versus Maintenance Dosing and Adverse Outcomes for Various Study Designs. **Table S4.** Sensitivity Analyses of Hazard Ratios to the Inclusion of Additional Covariates. **Table S5.** Characteristics of the Unweighted and Weighted Samples. **Table S6.** Multivariable-Adjusted and IPTW1-Adjusted Associations Between Bolus Versus Maintenance (Referent) Dosing and Study Outcomes (N=48,050). (PDF 3 MB)
