# Supplementary material for: Comparative short-term safety of bolus versus maintenance iron dosing in hemodialysis patients: a replication study
Source: BMC Nephrol. 2014 Sep 22;15:154. doi: 10.1186/1471-2369-15-154 (PMC4182851; doi:10.1186/1471-2369-15-154)
Supplement: Supplementary file 1 — Additional file 1: Table S1: Adverse Study Outcomes. Table S2. Definition of Covariates. Table S3. Unadjusted and Multivariable Adjusted Associations1 Between Bolus versus Maintenance Dosing and Adverse Outcomes for Various Study Designs. Table S4. Sensitivity Analyses of Hazard Ratios to the Inclusion of Additional Covariates. Table S5. Characteristics of the Unweighted and Weighted Samples. Table S6. Multivariable-Adjusted and IPTW1-Adjusted Associations Between Bolus Versus Maintenance (Referent) Dosing and Study Outcomes (N=48,050). (PDF 3 MB) [file 12882_2014_845_MOESM1_ESM.pdf]

Table S1 – Adverse Study Outcomes

Outcome	Definition	Data Source
Primary Outcomes		
All-cause death	Death as indicated in CMS file	CMS death notification file
CVD death	Primary cause of death: 23,26, 28, 29,36	CMS death notification file
Infection-related death	Primary cause of death: 33,34,45-48, 51,52,61-63,70	CMS death notification file
Hospitalized for stroke	Any ICD-9-CM diagnostic code of 435.xx, 436.xx, 433.x1, 434.x1, 437.1x, 437.9x	CMS Part A claims
Hospitalized for myocardial infarction	Primary ICD-9-CM code of 410.ss, hospital stay 3-180 days or died within 3 days	CMS Part A claims
Hospitalized for pneumonia, sepsis, vascular access infection	Any ICD-9-CM diagnostic codes of 996.62 (vascular access), 481.xx (pneumonia), 038.xx (sepsis)	CMS Part A claims
Hospitalized for infection (all major organ systems)	Any hospital admission with one of the following ICD-9-CM diagnostic codes as the principal diagnostic code: 001–139, 254.1, 320–326, 331.81, 372–372.39, 373.0–373.2, 382–382.4, 383.0, 386.33, 386.35, 388.60, 390–393, 421–421.1, 422.0, 422.91–422.93, 460–466, 472–474.0, 475–476.1, 478.21–478.24, 478.29, 480–490, 491.1, 494, 510–511, 513.0, 518.6, 519.01, 522.5, 522.7, 527.3, 528.3, 540–542, 566–567.9, 569.5, 572–572.1, 573.1–573.3, 575–575.12, 590–590.9, 595–595.4, 597–597.89, 598, 599.0, 601–601.9, 604–604.9, 607.1, 607.2, 608.0, 608.4, 611.0, 614–616.1, 616.3–616.4, 616.8, 670, 680–686.9, 706.0, 711–711.9, 730–730.3, 730.8–730.9, 790.7–790.8, 996.60–996.69, 997.62, 998.5, and 999.3.	CMS Part A claims
Use of IV antibiotics	Any indication of the use of the following drugs: Amikin® (amikacin sulfate); ampicillin; Ancef®, Kefzol® (cefazolin); aztreonam; Cefizox® (ceftizoxime); Cefotan® (cefotetan); Fortaz®, Tazicef® (ceftazidime); Claforan® (cefotaxime); clindamycin; Cubicin® (daptomycin); ethambutol; gentamicin; Keflin® (cephalothin); Levaquin® (levofloxacin); Mefoxin® (cefoxitin); Merrem® (meropenem); nafcillin; Nebcin® (tobramycin); oxacillin; Penicillin G; Zosyn® (piperacillin and tazobactam); Primaxin® (imipenem and cilastatin); Rocephin® (ceftriaxone); streptomycin; Timentin® (ticarcillin and clavulanate potassium); Unasyn® (ampicillin and sulbactam); Vancocin® (vancomycin); Vibramycin® (doxycycline); Zinacef® (cefuroxime); Zyvox® (linezolid)	Clinical Database

Table S2 – Definition of Covariates

COVARIATE	DEFINITION	SOURCE
Demographic		
Age	Categorized as: 16-45; 46-60; 61-75; >75 yrs.	USRDS
Sex	Male or female	USRDS
Race	White, Black, Other	USRDS
Clinical		
Vintage	Categorized as 0; 1-3; 4 or more yrs.	USRDS
BMI	Categorized as underweight, normal, overweight, obese	Clinical Database & USRDS
Anemia Management		
Access	Most recent vascular access (catheter vs fistula/graft) prior to TSAT index date	Clinical Database
EPO dose (baseline)	Total EPO dose, (quintiles)	Clinical Database
EPO dose (exposure)	Total EPO dose, (tertiles plus a no-use category)	Clinical Database
Index TSAT	Last TSAT at baseline (quintiles)	Clinical Database
Iron dose	Total dose at last month of baseline, categorized as none, low (1-200 mg), or high (>200mg)	Clinical Database
Hemoglobin	Most proximal Hb lab prior to index TSAT date (<10,10-11,>11-12,>12-13,>13)	Clinical Database
Ferritin	Most proximal serum ferritin prior to index TSAT date (quintiles)	Clinical Database
Albumin	At baseline (<3.3, 3.3-3.9, >3.9)	Clinical Database
Comorbidities		
Hospital days in last month of baseline	Categorized as 0, 1-3, <=4	USRDS, Medicare Part A Claims
Infection in last month	Any hospital admission in the last month with one of the following ICD-9-CM diagnostic codes as the principal diagnostic code: 001–139, 254.1, 320–326, 331.81, 372–372.39, 373.0–373.2, 382–382.4, 383.0, 386.33, 386.35, 388.60, 390–393, 421–421.1, 422.0, 422.91–422.93, 460–466, 472–474.0, 475–476.1, 478.21–478.24, 478.29, 480–490, 491.1, 494, 510–511, 513.0, 518.6, 519.01, 522.5, 522.7, 527.3, 528.3, 540–542, 566–567.9, 569.5, 572–572.1, 573.1–573.3, 575–575.12, 590–590.9, 595–595.4, 597–597.89, 598, 599.0, 601–601.9, 604–604.9, 607.1, 607.2, 608.0, 608.4, 611.0, 614–616.1, 616.3–616.4, 616.8, 670, 680–686.9, 706.0, 711–711.9, 730–730.3, 730.8–730.9, 790.7–790.8, 996.60–996.69, 997.62, 998.5, and 999.3.	USRDS, Medicare Part A Claims
	Any claims with the following HCPCS codes for antibiotic use in last month of baseline: J3370, J0690, J0713, J0692, J0696, J1580, J3260, J0278, J1840, J1956.	USRDS, Medicare Part A & B Claims
	Any indication of the use of the following drugs: Amikin® (amikacin sulfate); ampicillin; Ancef®, Kefzol® (cefazolin); aztreonam; Cefizox® (ceftizoxime); Cefotan® (cefotetan); Fortaz®, Tazicef® (ceftazidime); Claforan® (cefotaxime); clindamycin; Cubicin® (daptomycin); ethambutol; gentamicin; Keflin® (cephalothin); Levaquin® (levofloxacin); Mefoxin® (cefoxitin); Merrem® (meropenem); nafcillin; Nebcin® (tobramycin); oxacillin; Penicillin G; Zosyn® (piperacillin and tazobactam); Primaxin® (imipenem and cilastatin); Rocephin® (ceftriaxone); streptomycin; Timentin® (ticarcillin and	Clinical Database

	clavulanate potassium); Unasyn® (ampicillin and sulbactam); Vancocin® (vancomycin); Vibramycin® (doxycycline); Zinacef® (cefuroxime); Zyvox® (linezolid)	
Pneumonia	Any ICD-9-CM diagnostic code of 481.xx – 486.xx in baseline period	USRDS, Medicare Part A & B Claims
Vascular Access Infection	Any ICD-9-CM diagnostic code of 996.62 in baseline period	
Sepsis	Any ICD diagnostic code 038.xx, 995.90, 995.91, 995.92 in baseline period	
Diabetes	Any ICD-9-CM diagnostic code of 250.xx in baseline period	
Ischemic stroke	Any ICD-9-CM diagnostic code of 434.01, 434.11, 434.91, 435, 436, 437, 438, V12.54 in baseline period	
Myocardial infarction	Any ICD-9-CM diagnostic code of 410.xx in baseline period	
COPD	Any ICD-9-CM diagnostic code of 490.xx-496.xx, 505.xx, 506.4 in baseline period	
Cancer	Any ICD-9-CM diagnostic code of 173.3, 173.9, 174.0-175.9, 179-195, 196-199, 232.9, 233.0, 233.1, 300.29, 338.3, 789.51, 795.82, 799.4, V67.2, 200, 201, 202.0-202.3, 202.50-203.01,203.8, 238.6, 273.3 in baseline period	
Gastrointestinal bleeding	Any ICD-9-CM diagnostic code of 578.xx in baseline period	
Additional Comorbidities for Sensitivity Analyses		
Medicaid Eligibility	Indicator for dual eligibility during any part of the baseline	USRDS, Enrollment File
Year of treatment	2006, 2007, 2008, 2009, 2010	Clinical Database
Census Region	Based on location of last dialysis center in baseline period: Northeast, South, Midwest, West	USRDS, Enrollment File
ESRD Reason	Diabetes, Glomerulonephritis, hypertension, other	USRDS, Medical Evidence Form
Pulmonary circulation disease	Any ICD-9-CM diagnostic code of 415.xx-417.xx in baseline period	USRDS, Medicare Part A & B Claims
Peptic Ulcer Disease	Any ICD-9-CM diagnostic code of 530.2, 531.xx-534.xx, V12.71 in baseline period	
Liver disease	Any ICD-9-CM diagnostic code of 070.32, 070.33, 070.54, 456.0, 456.1, 456.20, 456.21, 571.0, 571.2, 571.3, 571.4, 571.5, 571.6, 571.8, 571.9, 572.3, 572.8, V42.7 in baseline period	
Other neurological problem	Any ICD-9-CM diagnostic code 331.9, 332.0, 333.4, 333.5, 334-335, 340, 341, 345.0, 345.1, 345.4, 345.5, 345.8, 345.9, 348.1, 348.3, 780.3, 784.3 in baseline period	
Substance abuse	Any ICD-9-CM diagnostic code 303.xx-305.xx in baseline period	
Ischemic Heart disease, other heart disease, peripheral vascular disease, history of CABG, Stent, PTCA	Any ICD-9-CM diagnostic code of 411.xx-414.xx, 420.xx-429.xx, 785.o, V45.0, v53.3, 402.11, 402.91, 404.11, 404.12, 404.91, 404.93, 093.2, 746.3-746.6, v42.2, v43.3, v43.4441.xx-443.xx, 447.1, 557.1, 557.9, 444.xx-445.xx; Procedure codes (both ICD-9-CM and CPT) of 00.66, 92982, 92985, 36.06, 36.07, 92980, 33510-33514, 33516-33519 in baseline period	
Hypertension	Any ICD-9-CM diagnostic code of 401.xx-405.xx, except 402.11, 402.91, 404.11, 404.13, 404.91, 404.93 in baseline period	
Rheumatic heart disease	Any ICD-9-CM diagnostic code of 393.xx -398.xx in baseline period	
Psychiatric problems	Any ICD-9-CM diagnostic code 295.xx-298.xx in baseline period	
Autoimmune disorders	Any ICD-9-CM diagnostic code of 564.1, 696.0, 696.1, 695.4,	

	710.0, 701.0, 710, 714, 720, 725 in baseline period	
Blood loss anemia	Any ICD-9-CM diagnostic code of 280.0 in baseline period	
Transfusion	Indicator for receipt of one or more transfusions during the baseline period, based on HCPCS codes P9010, P9011, P9016, P9021, P9022, P9038, P9039, P9040, 36430 and ICD-9 codes 99.03, 99.04	

Table S3 – Unadjusted and Multivariable Adjusted Associations[1] Between Bolus versus Maintenance Dosing and Adverse Outcomes for Various Study Designs

Design	Model	All Cause Death	Hospitalized pneumonia, vascular access infection (VAI), sepsis	Intravenous Antibiotics	Infection of any major organ system	Infection-related Death	Hospitalized pneumonia, (VAI), sepsis or infection death	Any infection or intravenous antibiotic use	Cardiovascular disease (CVD) death	Hospitalized Myocardial Infarction (MI)	Hospitalized Stroke	MI, Stroke or CVD death
**1 month/ 3 month**	Unadjusted	1.26 (1.11, 1.44)	1.41 (1.26, 1.58)	1.32 (1.23, 1.41)	1.43 (1.31, 1.56)	1.55 (1.09, 2.21)	1.42 (1.27, 1.58)	1.31 (1.24, 1.39)	1.08 (0.88, 1.34)	1.10 (0.83, 1.48)	1.24 (0.92, 1.66)	1.14 (0.99, 1.33)
	Adjusted	0.92 (0.79, 1.06)	1.08 (0.96, 1.21)	1.09 (1.01, 1.17)	1.13 (1.03, 1.24)	1.17 (0.80, 1.71)	1.08 (0.96, 1.21)	1.08 (1.02, 1.15)	0.82 (0.66, 1.02)	0.95 (0.69, 1.32)	1.07 (0.77, 1.47)	0.92 (0.78, 1.09)
**1 month/ 6 weeks**	Unadjusted	1.42 (1.17, 1.73)	1.48 (1.27, 1.74)	1.31 (1.20, 1.43)	1.43 (1.27, 1.61)	1.63 (0.90, 2.95)	1.48 (1.27, 1.73)	1.33 (1.24, 1.42)	1.31 (0.98, 1.74)	1.09 (0.72, 1.63)	1.12 (0.73, 1.73)	1.21 (0.98, 1.49)
	Adjusted	1.01 (0.81, 1.25)	1.09 (0.92, 1.28)	1.05 (0.96, 1.15)	1.11 (0.98, 1.26)	1.25 (0.65, 2.39)	1.09 (0.92, 1.28)	1.07 (1.00, 1.15)	1.01 (0.75, 1.36)	1.02 (0.65, 1.58)	1.00 (0.62, 1.61)	1.01 (0.80, 1.27)
**1 week/ 6 weeks**	Unadjusted	1.42 (1.12, 1.81)	1.73 (1.47, 2.05)	1.33 (1.20, 1.47)	1.58 (1.38, 1.80)	1.74 (0.91, 3.31)	1.72 (1.46, 2.03)	1.39 (1.28, 1.51)	1.07 (0.74, 1.54)	1.13 (0.71, 1.81)	0.89 (0.51, 1.52)	1.06 (0.81, 1.37)
	Adjusted	0.99 (0.77, 1.27)	1.21 (1.01, 1.45)	1.01 (0.90, 1.12)	1.14 (0.99, 1.31)	0.99 (0.50, 1.95)	1.20 (1.01, 1.44)	1.06 (0.98, 1.16)	0.80 (0.55, 1.17)	0.93 (0.56, 1.54)	0.65 (0.37, 1.13)	0.81 (0.61, 1.06)
**2 weeks/ 6 weeks**	Unadjusted	1.30 (1.05, 1.60)	1.75 (1.50, 2.04)	1.27 (1.15, 1.39)	1.57 (1.40, 1.77)	1.78 (1.04, 3.06)	1.75 (1.51, 2.04)	1.34 (1.25, 1.44)	1.07 (0.78, 1.49)	1.25 (0.85, 1.85)	1.15 (0.73, 1.81)	1.16 (0.93, 1.45)
	Adjusted	0.96 (0.77, 1.20)	1.21 (1.03, 1.43)	0.99 (0.90, 1.10)	1.16 (1.03, 1.31)	1.19 (0.66, 2.13)	1.21 (1.03, 1.43)	1.05 (0.97, 1.13)	0.83 (0.59, 1.15)	1.04 (0.68, 1.59)	0.98 (0.61, 1.57)	0.94 (0.74, 1.18)

[1] hazard ratios

Table S4 – Sensitivity Analyses of Hazard Ratios to the Inclusion of Additional Covariates

Design	Model	All Cause Death	Hospitalized pneumonia, vascular access infection (VAI), sepsis	Intravenous Antibiotics	Infection of any major organ system	Infection-related Death	Hospitalized pneumonia, (VAI), sepsis or infection death	Any infection or intravenous antibiotic use	Cardiovascular disease (CVD) death	Hospitalized Myocardial Infarction (MI)	Hospitalized Stroke	MI, Stroke or CVD death
**1 month/ 3 month**	Primary	0.92 (0.79, 1.06)	1.08 (0.96, 1.21)	1.09 (1.01, 1.17)	1.13 (1.03, 1.24)	1.17 (0.80, 1.71)	1.08 (0.96, 1.21)	1.08 (1.02, 1.15)	0.82 (0.66, 1.02)	0.95 (0.69, 1.32)	1.07 (0.77, 1.47)	0.92 (0.78, 1.09)
	Extended	0.90 (0.77, 1.04)	1.07 (0.95, 1.21)	1.09 (1.02, 1.18)	1.12 (1.02, 1.23)	1.18 (0.80, 1.76)	1.08 (0.96, 1.22)	1.07 (1.01, 1.14)	0.83 (0.66, 1.04)	0.98 (0.71, 1.37)	1.04 (0.75, 1.45)	0.94 (0.79, 1.10)
**1 month/ 6 weeks**	Primary	1.01 (0.81, 1.25)	1.09 (0.92, 1.28)	1.05 (0.96, 1.15)	1.11 (0.98, 1.26)	1.25 (0.65, 2.39)	1.09 (0.92, 1.28)	1.07 (1.00, 1.15)	1.01 (0.75, 1.36)	1.02 (0.65, 1.58)	1.00 (0.62, 1.61)	1.01 (0.80, 1.27)
	Extended	1.01 (0.81, 1.25)	1.06 (0.90, 1.26)	1.05 (0.96, 1.15)	1.09 (0.96, 1.24)	1.31 (0.66, 2.58)	1.07 (0.90, 1.26)	1.06 (0.99, 1.14)	1.04 (0.77, 1.41)	1.04 (0.67, 1.62)	1.03 (0.63, 1.69)	1.04 (0.83, 1.32)
**1 week/ 6 weeks**	Primary	0.99 (0.77, 1.27)	1.21 (1.01, 1.45)	1.01 (0.90, 1.12)	1.14 (0.99, 1.31)	0.99 (0.50, 1.95)	1.20 (1.01, 1.44)	1.06 (0.98, 1.16)	0.80 (0.55, 1.17)	0.93 (0.56, 1.54)	0.65 (0.37, 1.13)	0.81 (0.61, 1.06)
	Extended	0.97 (0.75, 1.26)	1.20 (1.00, 1.44)	1.00 (0.89, 1.11)	1.09 (0.95, 1.26)	1.03 (0.52, 2.06)	1.19 (1.00, 1.43)	1.02 (0.93, 1.11)	0.85 (0.58, 1.25)	0.96 (0.57, 1.61)	0.61 (0.35, 1.08)	0.83 (0.63, 1.09)
**2 weeks/ 6 weeks**	Primary	0.96 (0.77, 1.20)	1.21 (1.03, 1.43)	0.99 (0.90, 1.10)	1.16 (1.03, 1.31)	1.19 (0.66, 2.13)	1.21 (1.03, 1.43)	1.05 (0.97, 1.13)	0.83 (0.59, 1.15)	1.04 (0.68, 1.59)	0.98 (0.61, 1.57)	0.94 (0.74, 1.18)
	Extended	0.96 (0.77, 1.20)	1.21 (1.02, 1.43)	1.00 (0.90, 1.10)	1.14 (1.01, 1.29)	1.22 (0.67, 2.22)	1.21 (1.02, 1.43)	1.04 (0.97, 1.13)	0.85 (0.61, 1.19)	1.09 (0.71, 1.68)	0.97 (0.60, 1.57)	0.97 (0.76, 1.23)

Table S5 – Characteristics of the Unweighted and Weighted Samples

Characteristics<br>mean (SD) or %		Maintenance (unweighted)	Bolus (unweighted)	Maintenance (weighted)	Bolus (weighted)	Absolute Standardized Difference (weighted)
Age, y		61.4 (15.1)	60.1 (15.3	61.1 (15.2)	61.3 (15.1)	0.01
Female		45.1%	45.8%	45.3%	46.7%	0.03
Race:	Black race (ref=White)	54.1%	56.7%	54.7%	53.1%	0.03
	Other race (ref=White)	5.7%	5.1%	5.6%	5.6%	0.00
Vintage, y		5.1 (5.0)	4.8 (4.7)	5.0 (5.0)	4.8 (4.)	0.04
Body Mass Index		33.9 (29.8)	32.8 (23.5)	34.0 (29.7)	33.0 (24.0)	0.04
Catheter Use		19.5%	21.6%	20.1%	20.9%	0.02
Albumin at baseline		3.9 (0.4)	3.8 (0.4)	3.9 (0.4)	3.9 (0.4)	0.02
Hemoglobin at baseline		12.0 (1.3)	11.5 (1.4)	11.9 (1.3)	11.8 (1.3)	0.01
Index TSAT		31.4 (10.9)	23.7 (9.5)	29.8 (10.9)	29.2 (11.8)	0.05
Ferritin at baseline		745.5 (536.2)	624.6 (478.9)	723.3 (528.5)	723.1 (515.6)	0.00
Iron (mg) at baseline		279.1 (214.3)	313.6 (315.7)	283.4 (233.4)	321.0 (272.7)	0.15
EPO at baseline (1000U)		75.3 (78.3)	110.9 (98.2)	83.5 (84.2)	86.1 (84.9)	0.04
EPO during exposure (1000U)		73.1 (77.0)	113.2 (100.4)	81.9 (84.3)	85.2 (84.4)	0.04
Hospital days in last month		0.6 (1.9)	1.0 (2.3)	0.7 (2.0)	0.7 (2.0)	0.02
Infection in last month		12.6%	17.5%	13.8%	14.2%	0.01
Infection in last 6 mos:	Pneumonia	12.8%	16.7%	13.8%	14.3%	0.01
	Sepsis	19.0%	23.5%	20.2%	22.0%	0.04
	Vascular access	10.1%	15.0%	11.3%	11.7%	0.01
Diabetes		64.1%	67.5%	64.9%	64.9%	0.00
Ischemic stroke		12.9%	16.7%	13.8%	13.9%	0.00
Myocardial Infarction		4.4%	6.2%	4.7%	5.0%	0.01
COPD, Asthma		20.0%	24.4%	21.1%	21.7%	0.01
Cancer		10.9%	12.1%	11.1%	11.0%	0.00
GI bleeding		6.1%	9.5%	6.8%	6.8%	0.00

Table S6: Multivariable-Adjusted and IPTW[1]-Adjusted Associations Between Bolus Versus Maintenance (Referent) Dosing and Study Outcomes (N=48,050)

	Full Sample		Catheter Users	
	Hazard Ratio (95% CI)		Hazard Ratio (95% CI)	
**Outcome**	**Multivariable-Adjusted**	**IPTW-Adjusted**	**Multivariable-Adjusted**	**IPTW-Adjusted**
Death from any cause	0.92 (0.79, 1.06)	0.95 (0.80, 1.13)	1.10 (0.85, 1.43)	0.96 (0.72, 1.27)
Hospitalized for pneumonia, sepsis, vascular access infection	1.08 (0.96, 1.21)	1.05 (0.91, 1.21)	1.19 (0.99, 1.44)	1.24 (1.00, 1.53)
Intravenous Antibiotic Use	1.09 (1.01, 1.17)	1.10 (1.01, 1.20)	1.08 (0.96, 1.22)	1.16 (0.99, 1.35)
Hospitalized for infection of any major organ system	1.13 (1.03, 1.24)	1.15 (1.03, 1.29)	1.15 (0.98, 1.35)	1.18 (0.97, 1.43)
Infection-related death	1.17 (0.80, 1.71)	1.17 (0.77, 1.79)	1.39 (0.74, 2.61)	1.02 (0.54, 1.91)
Hospitalized for pneumonia, vascular access infection, sepsis or infection-related death	1.08 (0.96, 1.21)	1.05 (0.92, 1.21)	1.20 (0.99, 1.44)	1.23 (1.00, 1.51)
Hospitalized for any infection or intravenous antibiotic use	1.08 (1.02, 1.15)	1.08 (1.01, 1.17)	1.07 (0.97, 1.19)	1.12 (0.98, 1.28)
Hospitalized for stroke	1.07 (0.77, 1.47)	1.09 (0.75, 1.59)	1.30 (0.72, 2.35)	1.32 (0.71, 2.44)
Hospitalized for Myocardial Infarction	0.95 (0.69, 1.32)	1.11 (0.75, 1.62)	1.24 (0.66, 2.31)	1.40 (0.71, 2.77)
Cardiovascular-related death	0.82 (0.66, 1.02)	0.78 (0.59, 1.02)	1.14 (0.76, 1.73)	0.97 (0.60, 1.58)
Cardiovascular-related hospitalization or death	0.92 (0.78, 1.09)	0.95 (0.78, 1.16)	1.28 (0.94, 1.73)	1.26 (0.90, 1.76)

[1] inverse-probability of treatment weights
